# Accuracy of computed tomography perfusion-defined ischemic core and follow-up infarction after basilar artery thrombectomy

**DOI:** 10.3389/fneur.2025.1678450

**Published:** 2025-11-10

**Authors:** Pengjun Chen, Xia Li, Yechao Huang, Junguo Hui, Jie Rao, Wenya Zhang, Lijun Shang, Xiao Chen, Ruijie Gao, Jinwei Zhou, Shuiwei Xia, Qiaoling Ding, Jiansong Ji

**Affiliations:** 1 Zhejiang Key Laboratory of Imaging and Interventional Medicine, The Fifth Affiliated Hospital of Wenzhou Medical University, Lishui Hospital of Zhejiang University, Lishui, China; 2 The Second School of Clinical Medicine, Zhejiang Chinese Medical University, Hangzhou, China; 3 Department of Neurology, The Fifth Affiliated Hospital of Wenzhou Medical University, Lishui Hospital of Zhejiang University, Lishui, China; 4 Department of Radiology, Jinyun County People's Hospital, Lishui, China; 5 Department of Radiology, Affiliated Hangzhou Xixi Hospital, Zhejiang University School of Medicine, Hangzhou, China

**Keywords:** basilar artery, computed tomography perfusion, stroke, ischemic core, thrombectomy

## Abstract

**Purpose:**

Accurate identification of computed tomography (CT) perfusion ischemic core in patients with basilar artery occlusion (BAO) on admission remains challenging despite its critical role in prognostic prediction and treatment decision-making. We aimed to define the optimal threshold for identifying the ischemic core by assessing agreement in lesion extent and spatial distribution using Syngo.via.

**Methods:**

We retrospectively analyzed 91 patients with BAO who achieved successful recanalization after endovascular thrombectomy at our center. The ischemic core was estimated using the following thresholds: cerebral blood flow (CBF) < 10 or 15 mL/100 g/min by Syngo.via, cerebral blood volume < 1.2 mL/100 mL by Syngo.via, and time to maximum > 10 s by RAPID. The Posterior Circulation Alberta Stroke Program Early CT Score was used to assess the extent of the infarction. Statistical analyses included the intraclass correlation coefficient (ICC) and receiver operating characteristic analyses.

**Results:**

The CBF < 10 mL/100 g/min threshold demonstrated good agreement in extent with follow-up infarction (ICC: 0.81 [95% confidence intervals 0.72–0.87]), with overestimation or underestimation being the most uncommon (*n* = 9). For the detection of midbrain, pontine, and cerebellar infarction, this threshold yielded the best performance with the area under the curve ranging from 0.79 (midbrain, 0.66–0.93; *p* < 0.001) to 0.90 (pons, 0.83–0.98; *p* < 0.001).

**Conclusion:**

In patients with BAO after successful recanalization, the optimal threshold for the ischemic core was a CBF < 10 mL/100 g/min. This threshold may serve as a reliable imaging biomarker, aiding in the prediction of tissue outcomes and treatment decision-making.

## 1 Introduction

Computed tomography perfusion (CTP) imaging parameters, particularly the CTP-defined ischemic core, are widely used for early detection, treatment selection, and outcome prediction in patients with anterior circulation large artery occlusion (1–3). Compared to the contralateral hemisphere, a relative cerebral blood flow (rCBF) < 30% best depicts the final infarct profile following intravenous thrombolysis, and a rCBF volume < 20% is the closest predictor of the final infarct volume after endovascular treatment (3–8). However, patients with basilar artery occlusion (BAO) were excluded from these trials. Because of the bilateral symmetric hypoperfusion or infarction associated with BAO (9–11), these conventional thresholds for identifying the ischemic core may not apply to patients with BAO. Consequently, a new ischemic core approach with an optimal threshold is required. Normal brain mixed cortical flow averages approximately 51 mL/100 g/min (12, 13). Therefore, we aimed to use the absolute value of CBF as an alternative to rCBF and hypothesized that CBF < 10 mL/100 g/min (rCBF < 20%) and CBF < 15 mL/100 g/min (rCBF < 30%) are promising thresholds for identifying ischemic cores in patients with BAO.

Moreover, the ischemic core estimation approach using a cerebral blood volume (CBV) threshold of < 1.2 mL/100 mL is recommended by the Siemens Syngo.via software (Siemens Healthcare, Erlangen, Germany) for anterior circulation strokes; however, no validation has been conducted in patients with BAO (14, 15). Yuen found that the perfusion deficit volume of time to maximum (Tmax) > 10 s, generated by RAPID software (iSchemaView, Menlo Park, CA, USA), exhibited a good correlation with the final infarct volume in patients with BAO who underwent successful reperfusion (16). We selected these two methods as potential alternative thresholds for identifying the ischemic core in patients with BAO stroke.

Furthermore, in contrast to the predominant importance of infarct size in anterior circulation strokes, the involvement of critical brain regions, such as the pons and midbrain, appears to be more crucial for predicting functional outcomes in patients with BAO due to a higher density of cranial nerve nuclei, neural pathways, and reticular formation (9, 17–19).

We aimed to determine the optimal ischemic core threshold applicable to patients with BAO by evaluating the agreement in the extent and spatial distribution between follow-up infarction and four commonly used core estimation approaches: CBF < 10 mL/100 g/min (rCBF < 20%) by Syngo.via, CBF < 15 mL/100 g/min (rCBF < 30%) by Syngo.via, CBV < 1.2 mL/100 mL by Syngo.via, and Tmax >10 s by RAPID.

## 2 Methods

### 2.1 Patients

Ethical approval was obtained from the Institutional Review Board of Lishui Central Hospital (approval number: 2022357), and the study complied with the guidelines of the Declaration of Helsinki. The review board waived the requirement for written informed consent because of the retrospective nature of the study.

We retrospectively analyzed the imaging data of consecutive patients with BAO who underwent endovascular thrombectomy (EVT) between January 2018 and March 2024 at our single-institution stroke center.

**Figure 1:**
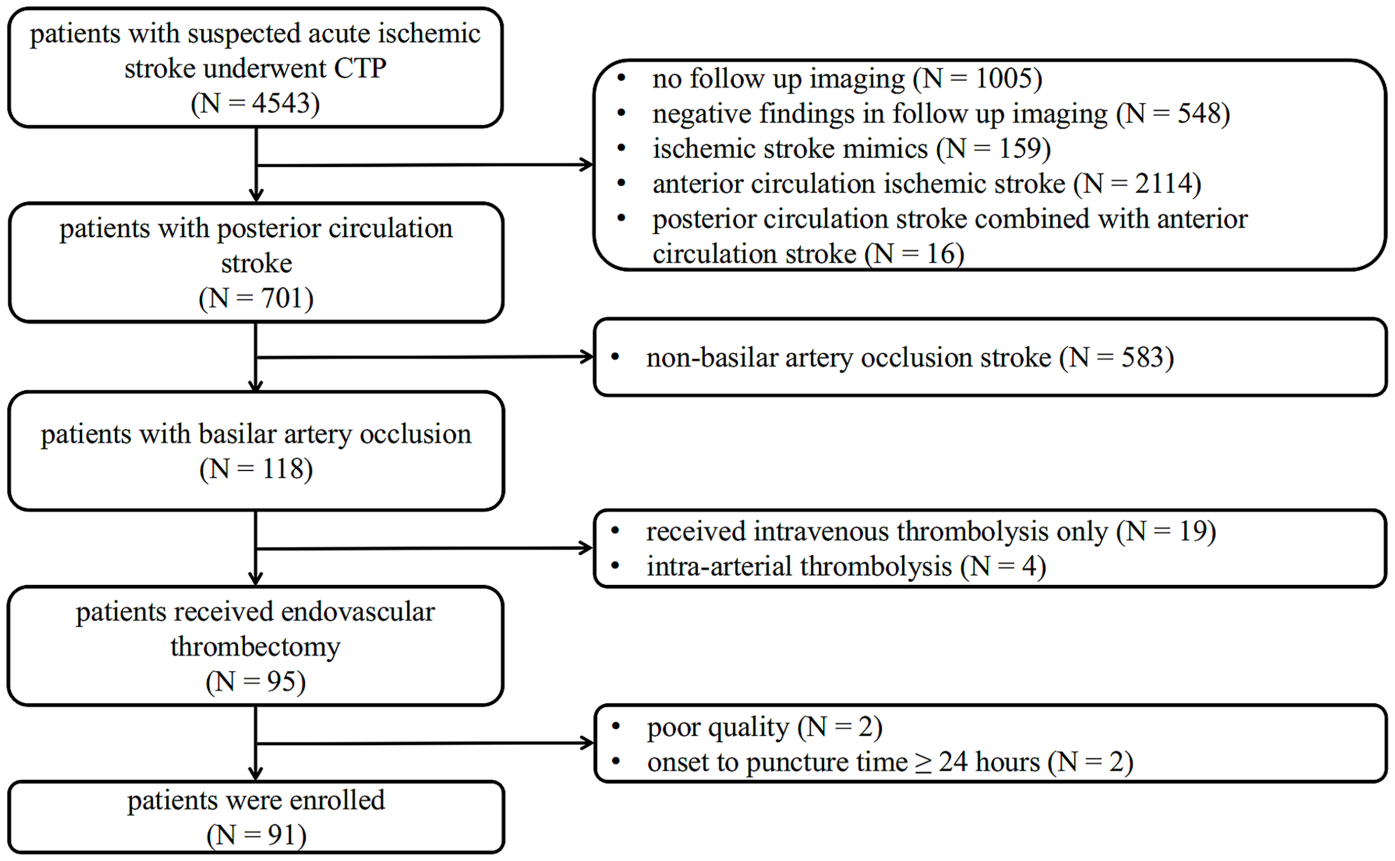
Flow chart of the study population. CTP, CT perfusion.

The inclusion criteria for eligible patients of this study were as follows: (1) baseline National Institutes of Health Stroke Scale (NIHSS) score ≥ 6; (2) a standard CT protocol covering noncontrast CT (NCCT) and CTP examination on admission, with significant perfusion deficits; (3) confirmed BAO on reconstructed CT Angiography (CTA); (4) execution of EVT; and (5) performance of MRI-diffusion weighted imaging (MRI-DWI) or NCCT as follow-up imaging within 3 days after EVT. The exclusion criteria were: (1) the time from symptom onset to EVT puncture ≥ 24 h; (2) stroke recurrence before follow-up image; and (3) non-interpretable CTP imaging due to poor quality. A flowchart of the study is shown in Figure 1.

A “significant perfusion deficit” was defined as a visual decrease in CBF or CBV or an increase in Tmax on at least two adjacent slices.

### 2.2 Imaging protocol

For all patients, a standardized CT protocol consisting of NCCT and CTP was performed from the base of the skull to the parietal lobe using SOMATOM Force scanners (Siemens Healthcare, Erlangen, Germany) on admission. NCCT imaging data (100 kVp, 310 mAs) were obtained with a slice thickness of 1 mm and reconstructed at 5 mm. CTP data were acquired every 1.5–3 s for 51 s after an initial 4-s delay. The scan was processed at 80 kVp and 100 mAs after intravenous injection of 50 mL contrast at 8 mL/s.

### 2.3 Data processing

All CTP raw data were transferred to Syngo.via CT Neuro Perfusion software version VB40 (Siemens Healthcare, Erlangen, Germany), and color-coded perfusion maps (including CBF, CBV, and Tmax) were obtained, with a slice thickness of 5 mm and reconstruction interval of 3 mm.

Four ischemic core estimation approaches generated by Syngo.via software or RAPID software were investigated. Approach 1 represents CBF < 10 mL/100 g/min (equivalent to rCBF < 20%) maps generated by Syngo.via software. Approach 2 represents CBF < 15 mL/100 g/min (equivalent to rCBF < 30%) maps generated by Syngo.via software. Approach 3 represents CBV < 1.2 mL/100 mL maps generated by Syngo.via software. Approach 4 represents Tmax > 10-s maps generated by RAPID software. We chose these approaches because they have been commonly used or recommended in prior studies and daily clinical practice.

For CTA analysis, slices were reconstructed at a thickness of 0.625 mm every 1 mm from the peak phase of the time-attenuation curve. CT attenuation was measured by placing a circular region of interest in the proximal middle cerebral artery on the axial images.

### 2.4 Baseline imaging assessment

Qualitative and semi-quantitative assessments of all imaging data were performed by two independent neuroradiologists (with 13 or 20 years of experience) who were blinded to both clinical data and follow-up imaging. In case of disagreement, a third neuroradiologist (with 25 years of experience) was invited, and the final decision was made in a separate session.

Location of the arterial occlusions was documented on CTA images. To evaluate early ischemic changes, NCCT, color-coded CTP maps, ischemic core maps from approaches 1 to 4, and follow-up imaging were assessed using the Posterior Circulation Alberta Stroke Program Early CT Score (pc-ASPECTS) (20). A lesion was considered positive if it had at least one diameter of 6 mm or more. The pc-ASPECTS is a 10-point score that decreases with the presence of ischemic changes in the following brain regions: 1 point is subtracted for the unilateral cerebellum, thalamus, or posterior cerebral artery (PCA) territory, and 2 points are deducted for the pons and midbrain. Ischemic changes were recorded in the pons, midbrain, unilateral thalamus, cerebellum, and the PCA territory, respectively.

Overestimations and underestimations were recorded using the pc-ASPECTS_difference_. The pc-ASPECTS_difference_ between the ischemic core estimation approach and follow-up infarction was calculated as follows:

$$\begin{array}{l} \mathrm{pc}-{\text{ASPECTS}}_{\text{difference}}=\mathrm{pc}-{\text{ASPECTS}}_{\text{follow}-\mathrm{up}\;\text{infarction}}\\ -\mathrm{pc}-{\text{ASPECTS}}_{\text{score}} \end{array}$$

A pc-ASPECTS_difference_ < −2 was defined as an underestimation of the ischemic core, and a pc-ASPECTS_difference_ > 2 was defined as an overestimation of the ischemic core.

### 2.5 Clinical data

Trained investigators were blinded to baseline and follow-up imaging. They collected the following clinical data: sex, age, baseline NIHSS score, onset-to-scan time, scan-to-recanalization time, and treatment data. Certified stroke neurologists performed NIHSS assessments as part of their routine clinical activities. Successful recanalization was defined as modified Thrombolysis in Cerebral Infarction (mTICI) 2b–3.

### 2.6 Statistical analysis

Statistical analyses were performed using MedCalc Statistical Software version 20.022 (MedCalc Software Ltd., Ostend, Belgium) and SPSS Statistics 27 (IBM Corp., Armonk, NY, USA). Continuous variables were described by mean ± standard deviation and compared by the Mann–Whitney U test. Categorical variables are expressed as counts and percentages and were compared using the chi-square test.

The pc-ASPECTS agreement between the CTP ischemic core estimation approaches and follow-up infarction was evaluated using the intraclass correlation coefficient (ICC) and the weighted Kappa coefficient. We based the ICC estimates on a two-way mixed model, absolute agreement, and average measures, and the degree of agreement was classified as previously suggested (ICC < 0.5 = poor agreement, ICC 0.5–0.75 = moderate agreement, ICC 0.75–0.9 = good agreement, and ICC > 0.9 = excellent agreement). Bland–Altman plots were also used to present the pc-ASPECTS agreement.

Receiver operating characteristic (ROC) analyses, including the area under the curve (AUC), sensitivity, and specificity, were performed to quantify the agreement of spatial distribution and the diagnostic accuracy across different brain regions.

Statistical significance was defined as *p* < 0.05.

## 3 Results

### 3.1 Study population

A flowchart of the study is shown in Figure 1. Between January 2018 and March 2024, 4,543 symptomatic patients with suspected acute ischemic stroke were referred for brain NCCT and CTP. Of these, 701 patients had a posterior circulation stroke diagnosed with follow-up imaging, and 118 patients were confirmed to have BAO. This study excluded 19 patients who underwent intravenous thrombolysis only, four patients who underwent intra-arterial thrombolysis, two patients with poor image quality, and two patients with an onset-to-puncture time of more than 24 h. A total of 91 patients (mean age 67.7 ± 11.2 years, 70.3% men) with BAO achieved successful recanalization after EVT and were enrolled.

The mean baseline NIHSS score was 22.7 ± 8.8. Thirty-nine (42.9%) patients underwent a CTP scan within 6 h after symptom onset, with a mean onset-to-scan time of 410.8 ± 219.1 min, ranging from 61 min to 1,138 min. Mean scan-to-recanalization time was 192.5 ± 38.6 min, ranging from 103 min to 272 min. Intravenous thrombolysis was administered to 20 (22.0%) patients before EVT at a primary or our stroke center. Of these, 61 (67.0%) patients were treated with EVT alone, while 30 (33.0%) patients received EVT combined with balloon angioplasty, stenting, or both. Of these, 80 (87.9%) patients achieved mTICI scores of 3, and 10 (12.1%) patients achieved mTICI scores of 2b. Of these 91 patients, 56 (61.5%) underwent MRI-DWI as follow-up imaging, and 35 (38.5%) underwent NCCT only due to contraindications. Mean pc-ASPECTS based on follow-up imaging was 5.9 ± 2.1 (Table 1).

**Table 1:** Patient characteristics.

Variables	Overall (*N* = 91)
Patient data	
Age, years	67.7 ± 11.2
Male sex, *n* (%)	64 (70.3%)
NIHSS score	22.7 ± 8.8
Onset-to-scan time within 6 h, *n* (%)	39 (42.9%)
Onset-to-scan time, min	410.8 ± 219.1
Scan-to-recanalization time, min	192.5 ± 38.6
Intravenous thrombolysis, *n* (%)	20 (22.0%)
EVT alone, *n* (%)	61 (67.0%)
mTICI 2b, *n* (%)	11 (12.1%)
mTICI 3, *n* (%)	80 (87.9%)
Follow up MRI-DWI, *n* (%)	56 (61.5%)
Imaging data	
pc-ASPECTS NCCT	8.2 ± 1.5
pc-ASPECTS CBF	3.6 ± 2.0
pc-ASPECTS CBV	7.1 ± 1.8
pc-ASPECTS Tmax	1.9 ± 2.3
pc-ASPECTS rCBF < 30% by Syngo.via	8.0 ± 1.6
pc-ASPECTS rCBF < 20% by Syngo.via	9.3 ± 1.1
pc-ASPECTS rCBF < 30% by RAPID	9.7 ± 0.6
pc-ASPECTS rCBF < 20% by RAPID	10.0 ± 0.1
pc-ASPECTS Approach 1	6.7 ± 2.0
pc-ASPECTS Approach 2	4.9 ± 2.3
pc-ASPECTS Approach 3	7.0 ± 2.1
pc-ASPECTS Approach 4	7.7 ± 2.3
pc-ASPECTS follow-up infarction	5.9 ± 2.1

Values presented are number (percentage) for categorical variables and mean for ordinal and continuous variables. NIHSS, National Institutes of Health Stroke Scale; EVT, endovascular thrombectomy; NCCT, noncontrast CT; mTICI, modified Thrombolysis In Cerebral Infarction; MRI-DWI, magnetic resonance imaging-diffusion weighted imaging; pc-ASPECTS, Posterior Circulation Alberta Stroke Program Early CT Score; CBF, cerebral blood flow; CBV, cerebral blood volume; Tmax, time to maximum.

Approach 1 represents CBF < 10 mL/100 g/min (rCBF < 20%) maps generated by Syngo.via software. Approach 2 represents CBF < 15 mL/100 g/min (rCBF < 30%) maps generated by Syngo.via software. Approach 3 represents CBV < 1.2 mL/100 mL maps generated by Syngo.via software. Approach 4 represents Tmax > 10 s maps generated by RAPID software.

### 3.2 Extensive agreement analysis

Among four approaches, the mean pc-ASPECTS_difference_ between CTP estimated ischemic core and follow-up infarction was as follows: approach 1: −0.9 ± 1.3, approach 2: 0.9 ± 1.8, approach 3: −1.2 ± 1.9, and approach 4: −1.8 ± 2.4. Compared with the follow-up infarction, CTP ischemic core overestimation for approaches 1 and 3 was the most uncommon (*n* = 0), and underestimation for approach 4 was the most common (*n* = 32). Notably, CTP ischemic core overestimation or underestimation for approach 1 was the most uncommon (*n* = 9) (Table 2).

**Table 2:** The pc-ASPECTS_difference_ between baseline imaging and follow-up infarction.

Variables	Overall (*N* = 91)	< −2 (*n*)	>2 (*n*)
pc-ASPECTS_difference_ NCCT	−2.4 ± 2.0	36	0
pc-ASPECTS_difference_ CBF	2.2 ± 1.9	0	35
pc-ASPECTS_difference_ CBV	1.2 ± 1.7	16	0
pc-ASPECTS_difference_ Tmax	3.9 ± 2.4	0	59
pc-ASPECTS_difference_ rCBF < 30% by Syngo.via	−1.9 ± 2.2	32	0
pc-ASPECTS_difference_ rCBF < 20% by Syngo.via	−3.3 ± 2.1	57	0
pc-ASPECTS_difference_ rCBF < 30% by RAPID	−3.7 ± 2.1	62	0
pc-ASPECTS_difference_ rCBF < 20% by RAPID	−4.0 ± 2.1	65	0
pc-ASPECTS_difference_ Approach 1	−0.9 ± 1.3	9	0
pc-ASPECTS_difference_ Approach 2	0.9 ± 1.8	3	15
pc-ASPECTS_difference_ Approach 3	−1.2 ± 1.9	16	0
pc-ASPECTS_difference_ Approach 4	−1.8 ± 2.4	32	5

NCCT, noncontrast CT; CBF, cerebral blood flow; CBV, cerebral blood volume; Tmax, time to maximum; pc-ASPECTS, Posterior Circulation Alberta Stroke Program Early CT Score.

Approach 1 represents CBF < 10 mL/100 g/min (rCBF < 20%) maps generated by Syngo.via software. Approach 2 represents CBF < 15 mL/100 g/min (rCBF < 30%) maps generated by Syngo.via software. Approach 3 represents CBV < 1.2 mL/100 mL maps generated by Syngo.via software. Approach 4 represents Tmax > 10 s maps generated by RAPID software.

ICC estimates showed poor to good agreement on lesion extent using pc-ASPECTS between ischemic core estimation approaches and follow-up infarction (approach 1: ICC 0.81 [95% confidence intervals (CI) 0.72–0.87], approach 2: ICC 0.64 [95% CI 0.51–0.75], approach 3: ICC 0.60 [95% CI 0.45–0.72], approach 4: ICC 0.42 [95% CI 0.23–0.57]). The weighted Kappa coefficient for all ischemic core estimation approaches was as follows: approach 1: 0.73 (0.63–0.83), approach 2: 0.62 (0.48–0.76), approach 3: 0.51 (0.36–0.67), and approach 4: 0.35 (0.20–0.50). Further details are provided in Table 3.

**Table 3:** The agreement in score assignment between baseline imaging and follow-up infarction.

Independent variables	Agreement		
	ICC	κ	*p* value
pc-ASPECTS NCCT	0.40 (0.22–0.56)	0.25 (0.13–0.37)	< 0.001*
pc-ASPECTS CBF	0.59 (0.43–0.71)	0.44 (0.31–0.56)	< 0.001*
pc-ASPECTS CBV	0.62 (0.48–0.73)	0.51 (0.38–0.65)	< 0.001*
pc-ASPECTS Tmax	0.37 (0.30–0.69)	0.19 (0.10–0.28)	< 0.001*
pc-ASPECTS Approach 1	0.81 (0.72–0.87)	0.73 (0.63–0.83)	< 0.001*
pc-ASPECTS Approach 2	0.64 (0.51–0.75)	0.62 (0.48–0.76)	< 0.001*
pc-ASPECTS Approach 3	0.60 (0.45–0.72)	0.51 (0.36–0.67)	< 0.001*
pc-ASPECTS Approach 4	0.42 (0.23–0.57)	0.35 (0.20–0.50)	< 0.001*

ICC, intraclass correlation coefficient; pc-ASPECTS, Posterior Circulation Alberta Stroke Program Early CT Score; NCCT, noncontrast CT; CBF, cerebral blood flow; CBV, cerebral blood volume; Tmax, time to maximum.

Approach 1 represents CBF < 10 mL/100 g/min (rCBF < 20%) maps generated by Syngo.via software. Approach 2 represents CBF < 15 mL/100 g/min (rCBF < 30%) maps generated by Syngo.via software. Approach 3 represents CBV < 1.2 mL/100 mL maps generated by Syngo.via software. Approach 4 represents Tmax > 10 s maps generated by RAPID software.

*p values indicate *p* < 0.05.

**Figure 2:**
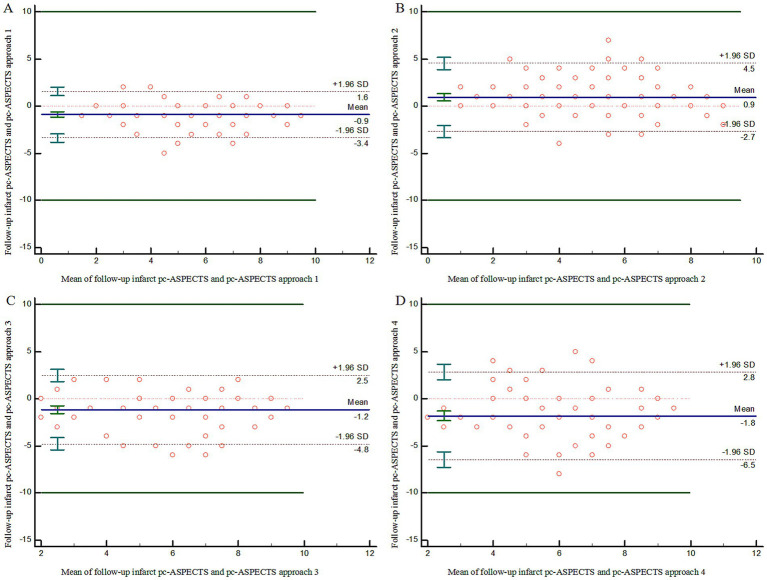
Bland–Altman Plot demonstrating agreement on lesion extent using pc-ASPECTS between follow-up infarction and ischemic core approach 1–4 **(A–D)**. pc-ASPECTS, Posterior Circulation Alberta Stroke Program Early CT Score; Approach 1 represents CBF < 10 mL/100 g/min (rCBF < 20%) maps generated by Syngo.via software. Approach 2 represents CBF < 15 mL/100 g/min (rCBF < 30%) maps generated by Syngo.via software. Approach 3 represents CBV < 1.2 mL/100 mL maps generated by Syngo.via software. Approach 4 represents Tmax > 10 s maps generated by RAPID software.

**Figure 3:**
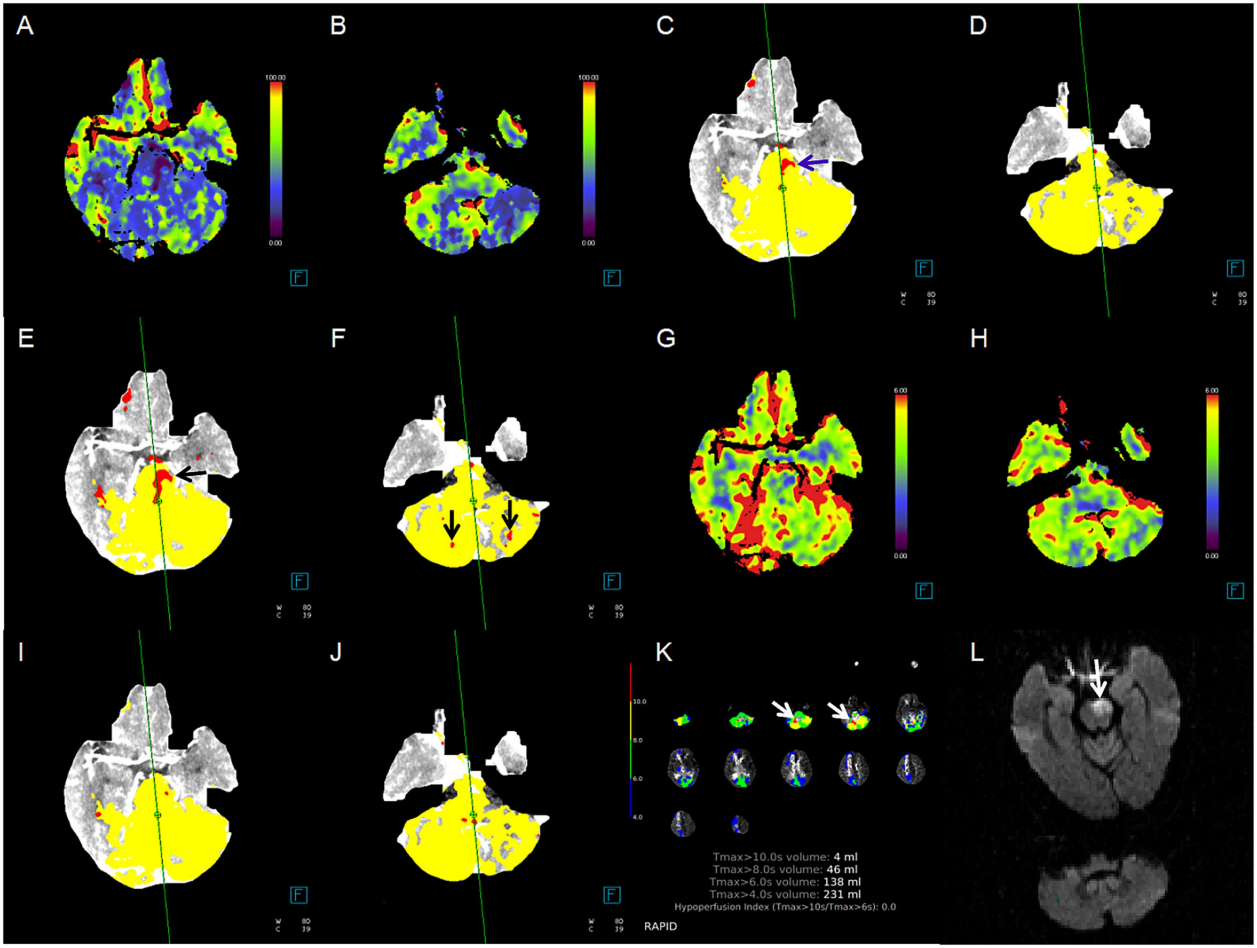
Case example of comparison between CTP ischemic core estimation approaches and follow-up infarction. Red area indicates the ischemic core. **(A,B)** Cerebral blood flow (CBF) maps, ischemic lesions in the pons and bilateral cerebellum; **(C,D)** ischemic core approach 1, ischemic lesions in the pons only (blue arrow); **(E,F)** ischemic core approach 2, ischemic lesions in the pons and bilateral cerebellum (black arrow); **(G,H)** cerebral blood volume (CBV) maps, none ischemic lesion; **(I,J)** ischemic core approach 3, none ischemic lesion; **(K)** ischemic core approach 4, ischemic lesions in right cerebellum only (white arrow); **(L)** follow-up MRI-DWI, pontine infarction (white arrow). Approach 1 represents CBF < 10 mL/100 g/min (rCBF < 20%) maps generated by Syngo.via software. Approach 2 represents CBF < 15 mL/100 g/min (rCBF < 30%) maps generated by Syngo.via software. Approach 3 represents CBV < 1.2 mL/100 mL maps generated by Syngo.via software. Approach 4 represents Tmax > 10 s maps generated by RAPID software.

Figure 2 illustrates the pc-ASPECTS agreement between ischemic core estimation approaches and follow-up infarction using the Bland–Altman plot; the positive bias indicated that follow-up infarction pc-ASPECTS was lower than the CTP-estimated ischemic core. Figure 3 shows a comparison between CTP ischemic core estimation approaches and follow-up infarction.

### 3.3 Spatial agreement analysis

In ROC analyses, for detection of midbrain, pontine, and cerebellar infarction, the ischemic core estimation approach (CBF < 10 mL/100 g/min by Syngo.via) yielded the best performance with the AUC ranging from 0.79 (midbrain infarction, 0.66–0.93; *p* < 0.001) to 0.90 (pontine infarction, 0.83–0.98; *p* < 0.001, with sensitivity and specificity of 90.16 and 90.00%, respectively). In the detection of unilateral thalamus and PCA territory stroke, ischemic core estimation approach 2 showed a good performance, with the most significant AUC of 0.79–0.84 (*p* < 0.001). Further details are provided in Table 4 and Supplementary Table 1.

**Table 4:** Receiver operating characteristics analysis of classification of infarction in different brain regions.

Variables	Follow-up infarction					
	Negative	Positive	Area under the curve	*p* value	Sensitivity	Specificity
Pons	30	61				
Approach 1	33	58	0.90 (0.83–0.98)	< 0.001*	90.16%	90.00%
Approach 2	22	69	0.74 (0.62–0.86)	< 0.001*	91.80%	56.67%
Approach 3	37	54	0.74 (0.63–0.86)	< 0.001*	75.41%	73.33%
Approach 4	61	30	0.65 (0.53–0.76)	0.02*	42.62%	86.67%
Midbrain	71	20				
Approach 1	78	13	0.79 (0.66–0.93)	< 0.001*	65.00%	100.00%
Approach 2	49	42	0.69 (0.56–0.81)	0.01*	75.00%	61.97%
Approach 3	78	13	0.73 (0.58–0.88)	< 0.01*	50.00%	95.77%
Approach 4	69	22	0.57 (0.42–0.72)	0.35	35.00%	78.87%
Right thalamus	66	25				
Approach 1	79	12	0.74 (0.61–0.87)	< 0.001*	48.00%	100.00%
Approach 2	75	16	0.79 (0.67–0.92)	< 0.001*	60.00%	98.48%
Approach 3	80	11	0.72 (0.59–0.86)	< 0.01*	44.00%	100.00%
Approach 4	87	4	0.55 (0.41–0.69)	0.44	12.00%	98.48%
Left thalamus	67	24				
Approach 1	82	9	0.75 (0.62–0.88)	< 0.01*	50.00%	100.00%
Approach 2	79	12	0.79 (0.67–0.91)	< 0.001*	66.67%	91.04%
Approach 3	80	11	0.73 (0.59–0.87)	< 0.01*	45.83%	100.00%
Approach 4	82	9	0.57 (0.43–0.72)	0.28	20.83%	94.03%
Right PCA territory	69	22				
Approach 1	77	14	0.79 (0.66–0.92)	< 0.001*	59.09%	98.55%
Approach 2	67	24	0.84 (0.73–0.95)	< 0.001*	77.27%	89.86%
Approach 3	78	13	0.77 (0.63–0.90)	< 0.001*	54.55%	98.55%
Approach 4	80	11	0.63 (0.48–0.78)	0.08	31.82%	94.20%
Left PCA territory	75	16				
Approach 1	79	12	0.80 (0.65–0.95)	< 0.001*	62.50%	97.33%
Approach 2	68	23	0.80 (0.67–0.93)	< 0.001*	75.00%	85.33%
Approach 3	81	10	0.74 (0.58–0.90)	< 0.01*	50.00%	97.33%
Approach 4	81	10	0.70 (0.53–0.86)	< 0.01*	43.75%	96.00%
Right cerebellum	29	62				
Approach 1	37	54	0.89 (0.81–0.96)	< 0.001*	83.87%	93.10%
Approach 2	14	77	0.64 (0.51–0.77)	0.03*	93.55%	34.48%
Approach 3	41	50	0.80 (0.70–0.90)	< 0.001*	74.19%	86.21%
Approach 4	53	38	0.68 (0.57–0.79)	< 0.01*	53.23%	82.76%
Left cerebellum	25	66				
Approach 1	42	49	0.82 (0.72–0.91)	< 0.001*	71.21%	92.00%
Approach 2	13	78	0.68 (0.54–0.81)	< 0.01*	95.45%	40.00%
Approach 3	48	43	0.74 (0.64–0.85)	< 0.001*	60.61%	88.00%
Approach 4	55	36	0.66 (0.54–0.78)	0.02*	48.48%	84.00%

PCA, posterior cerebral artery.

Approach 1 represents CBF < 10 mL/100 g/min (rCBF < 20%) maps generated by Syngo.via software. Approach 2 represents CBF < 15 mL/100 g/min (rCBF < 30%) maps generated by Syngo.via software. Approach 3 represents CBV < 1.2 mL/100 mL maps generated by Syngo.via software. Approach 4 represents Tmax > 10 s maps generated by RAPID software.

*p values indicate *p* < 0.05.

## 4 Discussion

Our study showed good agreement on the extent of the CTP-defined ischemic core with a threshold of CBF < 10 mL/100 g/min using Syngo.via software and follow-up infarction in patients with BAO who underwent successful recanalization. Notably, this method exhibits superior spatial diagnostic accuracy for the early detection of infratentorial infarctions, particularly pontine infarctions, and outperforms other threshold estimation approaches and color-coded perfusion maps. However, to identify stroke in the thalamus and PCA territory, a CBF threshold of < 15 mL/100 g/min for the ischemic core showed optimal classification performance.

Accurate estimation of the ischemic core has the potential to inform clinical therapy decision-making and predict functional outcomes. Compared with the conventional rCBF-based threshold of less than 30%, a stricter rCBF-based threshold for ischemic core estimation provides greater accuracy in patients with large artery occlusion after rapid recanalization (3, 4, 8, 21). Sarraj found that the ischemic core volume with a threshold of rCBF < 20% was comparable to and did not overestimate the final infarct volume in patients with an onset-to-scan time within 90 min after rapid reperfusion (4). However, no validation studies have been conducted in patients with BAO. The primary reason is that bilateral symmetric hypoperfusion changes or infarctions are associated with BAO, which can lead to inaccuracies in the rCBF-based ischemic core (10). This study aimed to determine the optimal threshold for the ischemic core estimation in patients with BAO. Our findings suggest that a conservative absolute value of the CBF-based threshold is more accurate in patients with BAO after successful reperfusion, particularly in those with infratentorial infarction.

To assess the extent of lesions in patients with anterior circulation large-artery occlusion, the infarct size is the primary comparison method (4, 6, 14, 22, 23). Based on voxel-based analysis and volume-based analysis, Edwards et al. (24) found that the optimal threshold was a mean transit time > 165%. However, the situation varies in the case of BAO. The density and distribution of neural pathways and nuclei across different posterior circulation brain regions indicate that lesion location may be more critical than infarct volume (11). For instance, patients with a smaller infarction in the pons or midbrain may have a poorer prognosis than those with a larger infarction in the cerebellum or occipital lobe. Consequently, several scoring systems have been developed to evaluate patients with BAO, including the pc-ASPECTS (20, 25), pons–midbrain index (26), pons–midbrain and thalamus scores (18), and the critical area perfusion score (9). Among these, pc-ASPECTS is the most widely used (24, 27, 28). Compared with other scoring systems, such as PMI, PMT, and CAPS, the pc-ASPECTS stands out by encompassing all brain regions within the posterior circulation except the medulla oblongata, including the pons, midbrain, bilateral cerebellum, thalamus, and the territory of the posterior cerebral artery (PCA). It is the most comprehensive method for assessing the affected areas in posterior circulation infarction. The pc-ASPECTS based on NCCT and CTA source images is an independent predictor of prognosis in patients with posterior circulation infarctions (20). Alemseged extends their works and found that pc-ASPECTS based on color-coded CTP maps has a higher predictive value for prognosis, with a CBV pc-ASPECTS ≤ 8 indicating a poor prognosis (29). However, this qualitative visual inspection method is time-consuming and highly dependent on the observer’s ability. A study by Edwards et al. (30) demonstrated that the optimal threshold for identifying the ischemic core in acute posterior circulation infarction was a delayed time exceeding 1.5 s. Nevertheless, it was observed that the assessment of the ischemic core within the basilar perforating arteries and the thalamus was notably inaccurate. Few studies have explored the correlation between the ischemic core and follow-up infarction in patients with BAO. Based on previous studies, we used pc-ASPECTS to assess the early ischemic extent. We discovered that the ischemic core defined by CTP, utilizing a CBF threshold of <10 mL/100 min set by Syngo via software, exhibited the least tendency to overestimate or underestimate follow-up infarction compared with NCCT, color-coded maps, and other threshold cores.

Pixel-to-pixel Dice coefficients have been utilized in previous studies to evaluate the spatial agreement in patients with anterior circulation strokes. However, the agreement was poor, with values ranging from 0.12 to 0.21 (4, 6, 14). Considering the diverse predictive values observed in different posterior circulation brain regions, we assessed the diagnostic accuracy of baseline imaging in detecting infarctions in all brain regions. Our results suggest that an ischemic core with a CBF threshold of < 10 mL/100 g/min generated by Syngo.via software provides valuable information, particularly for visualizing infratentorial infarcts.

Despite the availability of numerous commercial perfusion software packages from different vendors, none have been recognized as a standard strategy specifically suited for posterior circulation assessments (15, 31–33). RAPID software is currently the most widely used commercial package in clinical practice. However, Chen explored 116 patients with isolated pontine or midbrain hypoperfusion, of which 113 cases were confirmed as infarction on follow-up MRI. None of these lesions could be detected by the RAPID software (34), suggesting the potential for underestimation of infarction. Our results also demonstrated poor agreement in pc-ASPECTS scores and spatial distribution between Tmax > 10-s maps generated by the RAPID software and follow-up infarctions, suggesting that the RAPID software has low sensitivity when identifying pontine or midbrain infarctions.

In our cohort, for infarctions in the thalamus and posterior cerebral artery regions, the diagnostic efficacy of rCBF < 20% was inferior to that of rCBF < 30% and CBF-based CTP color-coded maps. This could be attributed to the compensatory effect of the posterior communicating artery, which may delay the decrease in CBF and reduce the sensitivity of the CBF-based ischemic core in these regions (35, 36).

### 4.1 Limitations

Our study had some limitations. First, this was a retrospective study conducted at a single comprehensive stroke center with a moderate sample size. Therefore, external validation with a larger sample size is required to confirm our findings. Second, our imaging protocol utilized a single CT scanner, which limits the transferability of our results to different vendors and centers. Further validation across multiple centers and CT scanners is required. Third, the optimal threshold for BAO may differ from that used for anterior circulation infarctions. However, owing to limited available evidence, the promising threshold candidates selected in our study were based on the anterior circulation in previous studies.

## 5 Conclusion

In patients with BAO after successful recanalization, the CTP ischemic core estimation approach with a CBF-based threshold of absolute value < 10 mL/100 g/min (rCBF < 20%) demonstrated good agreement between the lesion extent and spatial distribution with follow-up infarction. This method exhibited superior diagnostic accuracy for the detection and localization of infratentorial infarctions, particularly pontine infarctions.

## Data Availability

The raw data supporting the conclusions of this article will be made available by the authors, without undue reservation.
