# Supplementary material for: Accuracy of computed tomography perfusion-defined ischemic core and follow-up infarction after basilar artery thrombectomy
Source: Front Neurol. 2025 Nov 10;16:1678450. doi: 10.3389/fneur.2025.1678450 (PMC12641287; doi:10.3389/fneur.2025.1678450)
Supplement: Supplementary file 1 [file Table_1.docx]

Accuracy of computed tomography perfusion-defined ischemic core and follow-up infarction after basilar artery thrombectomy

Supplemental Material

Supplemental Table 1: Receiver operating characteristics analysis of classification of infarction in different brain regions (full version)

| Variables | follow-up infarction | | | | | |
| --- | --- | --- | --- | --- | --- | --- |
|  | Negative | Positive | area under the curve | *P* value | Sensitivity | Specificity |
| pons | 30 | 61 |  |  |  |  |
| NCCT | 74 | 17 | 0.61 (0.50-0.73) | 0.08 | 26.23% | 96.67% |
| CBF | 16 | 75 | 0.72 (0.59-0.84) | < 0.01* | 96.72% | 46.67% |
| CBV | 48 | 43 | 0.83 (0.74-0.91) | < 0.001* | 68.85% | 96.67% |
| Tmax | 11 | 80 | 0.63 (0.50-0.76) | 0.04* | 96.72% | 30.00% |
| Appoach 1 | 33 | 58 | 0.90 (0.83-0.98) | < 0.001* | 90.16% | 90.00% |
| Appoach 2 | 22 | 69 | 0.74 (0.62-0.86) | < 0.001* | 91.80% | 56.67% |
| Appoach 3 | 37 | 54 | 0.74 (0.63-0.86) | < 0.001* | 75.41% | 73.33% |
| Appoach 4 | 61 | 30 | 0.65 (0.53-0.76) | 0.02* | 42.62% | 86.67% |
| midbrain | 71 | 20 |  |  |  |  |
| NCCT | 90 | 1 | 0.53 (0.38-0.67) | 0.73 | 5.00% | 100.00% |
| CBF | 30 | 61 | 0.58 (0.45-0.72) | 0.26 | 80.00% | 36.62% |
| CBV | 81 | 10 | 0.69 (0.54-0.84) | 0.01* | 40.00% | 97.18% |
| Tmax | 12 | 79 | 0.55 (0.42-0.69) | 0.48 | 95.00% | 15.49% |
| Appoach 1 | 78 | 13 | 0.79 (0.66-0.93) | < 0.001* | 65.00% | 100.00% |
| Appoach 2 | 49 | 42 | 0.69 (0.56-0.81) | 0.01* | 75.00% | 61.97% |
| Appoach 3 | 78 | 13 | 0.73 (0.58-0.88) | < 0.01* | 50.00% | 95.77% |
| Appoach 4 | 69 | 22 | 0.57 (0.42-0.72) | 0.35 | 35.00% | 78.87% |
| Right thalamus | 66 | 25 |  |  |  |  |
| NCCT | 69 | 22 | 0.78 (0.65-0.90) | < 0.001* | 64.00% | 90.91% |
| CBF | 44 | 47 | 0.81 (0.72-0.90) | < 0.001* | 96.00% | 65.15% |
| CBV | 70 | 21 | 0.81 (0.69-0.93) | < 0.001* | 68.00% | 93.94% |
| Tmax | 32 | 59 | 0.69 (0.58-0.80) | < 0.01* | 92.00% | 45.45% |
| Appoach 1 | 79 | 12 | 0.74 (0.61-0.87) | < 0.001* | 48.00% | 100.00% |
| Appoach 2 | 75 | 16 | 0.79 (0.67-0.92) | < 0.001* | 60.00% | 98.48% |
| Appoach 3 | 80 | 11 | 0.72 (0.59-0.86) | < 0.01* | 44.00% | 100.00% |
| Appoach 4 | 87 | 4 | 0.55 (0.41-0.69) | 0.44 | 12.00% | 98.48% |
| Left thalamus | 67 | 24 |  |  |  |  |
| NCCT | 76 | 15 | 0.76 (0.62-0.89) | < 0.001* | 54.17% | 97.01% |
| CBF | 43 | 48 | 0.74 (0.63-0.85) | < 0.01* | 87.50% | 59.70% |
| CBV | 76 | 15 | 0.78 (0.66-0.91) | < 0.001* | 58.33% | 98.51% |
| Tmax | 28 | 63 | 0.68 (0.57-0.91) | < 0.01* | 95.83% | 40.30% |
| Appoach 1 | 82 | 9 | 0.75 (0.62-0.88) | < 0.01* | 50.00% | 100.00% |
| Appoach 2 | 79 | 12 | 0.79 (0.67-0.91) | < 0.001* | 66.67% | 91.04% |
| Appoach 3 | 80 | 11 | 0.73 (0.59-0.87) | < 0.01* | 45.83% | 100.00% |
| Appoach 4 | 82 | 9 | 0.57 (0.43-0.72) | 0.28 | 20.83% | 94.03% |
| Right PCA territory | 69 | 22 |  |  |  |  |
| NCCT | 82 | 9 | 0.68 (0.53-0.82) | 0.01* | 36.36% | 98.55% |
| CBF | 67 | 24 | 0.84 (0.73-0.95) | < 0.001* | 77.27% | 89.86% |
| CBV | 82 | 9 | 0.71 (0.56-0.85) | < 0.01* | 40.91% | 100.00% |
| Tmax | 32 | 59 | 0.73 (0.63-0.83) | < 0.01* | 100.00% | 46.38% |
| Appoach 1 | 77 | 14 | 0.79 (0.66-0.92) | < 0.001* | 59.09% | 98.55% |
| Appoach 2 | 67 | 24 | 0.84 (0.73-0.95) | < 0.001* | 77.27% | 89.86% |
| Appoach 3 | 78 | 13 | 0.77 (0.63-0.90) | < 0.001* | 54.55% | 98.55% |
| Appoach 4 | 80 | 11 | 0.63 (0.48-0.78) | 0.07 | 31.82% | 94.20% |
| Left PCA territory | 75 | 16 |  |  |  |  |
| NCCT | 81 | 10 | 0.78 (0.62-0.93) | < 0.01* | 56.25% | 98.67% |
| CBF | 73 | 18 | 0.84 (0.70-0.97) | < 0.001* | 75.00% | 92.00% |
| CBV | 83 | 8 | 0.71 (0.55-0.88) | < 0.01* | 43.75% | 98.67% |
| Tmax | 32 | 59 | 0.71 (0.60-0.83) | < 0.01* | 100.00% | 42.67% |
| Appoach 1 | 79 | 12 | 0.80 (0.65-0.95) | < 0.001* | 62.50% | 97.33% |
| Appoach 2 | 68 | 23 | 0.80 (0.67-0.93) | < 0.001* | 75.00% | 85.33% |
| Appoach 3 | 81 | 10 | 0.74 (0.58-0.90) | < 0.01* | 50.00% | 97.33% |
| Appoach 4 | 81 | 10 | 0.70 (0.53-0.86) | 0.01* | 43.75% | 96.00% |
| Right cerebellum | 29 | 62 |  |  |  |  |
| NCCT | 57 | 34 | 0.67 (0.56-0.79) | < 0.01* | 48.39% | 86.21% |
| CBF | 5 | 86 | 0.56 (0.43-0.69) | 0.37 | 98.39% | 13.79% |
| CBV | 33 | 58 | 0.74 (0.63-0.86) | < 0.001* | 79.03% | 68.97% |
| Tmax | 3 | 88 | 0.53 (0.40-0.66) | 0.70 | 98.39% | 6.90% |
| Appoach 1 | 37 | 54 | 0.89 (0.81-0.96) | < 0.001* | 83.87% | 93.10% |
| Appoach 2 | 14 | 77 | 0.64 (0.51-0.77) | 0.03* | 93.55% | 34.48% |
| Appoach 3 | 41 | 50 | 0.80 (0.70-0.90) | < 0.001* | 74.19% | 86.21% |
| Appoach 4 | 53 | 38 | 0.68 (0.57-0.79) | < 0.01* | 53.23% | 82.76% |
| Left cerebellum | 25 | 66 |  |  |  |  |
| NCCT | 57 | 34 | 0.65 (0.53-0.77) | 0.06 | 45.45% | 84.00% |
| CBF | 6 | 85 | 0.59 (0.45-0.73) | 0.07 | 98.48% | 20.00% |
| CBV | 40 | 51 | 0.72 (0.60-0.84) | 0.06 | 68.18% | 76.00% |
| Tmax | 3 | 88 | 0.56 (0.42-0.70) | 0.07 | 100.00% | 12.00% |
| Appoach 1 | 42 | 49 | 0.82 (0.72-0.91) | < 0.001* | 71.21% | 92.00% |
| Appoach 2 | 13 | 78 | 0.68 (0.54-0.81) | < 0.01* | 95.45% | 40.00% |
| Appoach 3 | 48 | 43 | 0.74 (0.64-0.85) | < 0.001* | 60.61% | 88.00% |
| Appoach 4 | 55 | 36 | 0.66 (0.54-0.78) | 0.02* | 48.48% | 84.00% |

Note: NCCT, noncontrast CT; CBF, cerebral blood flow; CBV, cerebral blood volume; Tmax, time to maximum; PCA, posterior cerebral artery.

Approach 1 represents CBF < 10 mL/100 g/min (rCBF < 20%) maps generated by Syngo.via software. Approach 2 represents CBF < 15 mL/100 g/min (rCBF < 30%) maps generated by Syngo.via software. Approach 3 represents CBV < 1.2 mL/100 mL maps generated by Syngo.via software. Approach 4 represents Tmax > 10 seconds maps generated by RAPID software.

**P* values indicate *P* < 0.05.
